# Molecular Mechanisms of *Nostoc flagelliforme* Environmental Adaptation: A Comprehensive Review

**DOI:** 10.3390/plants14111582

**Published:** 2025-05-22

**Authors:** Jin-Long Shang, Yong-Xue Xie, Lu-Yao Shi, Shuo-Ren Diao, Jin-Yan Guan

**Affiliations:** 1 Xinjiang Key Laboratory of Special Species Conservation and Regulatory Biology, College of Life Science, Xinjiang Normal University, Urumqi 830054, China; m13999125934@126.com (Y.-X.X.); 18803451367@163.com (L.-Y.S.); 17304387825@163.com (S.-R.D.); 2 College of Chemistry and Chemical Engineering, Xinjiang Normal University, Urumqi 830054, China

**Keywords:** desiccation tolerance, cyanobacteria, photoprotection, stress response regulation, *Nostoc flagelliforme*

## Abstract

*Nostoc flagelliforme*, a filamentous cyanobacterium inhabiting desert biological soil crusts (BSCs), has developed exceptional strategies to endure extreme environmental stresses, including severe desiccation, intense ultraviolet (UV) radiation, and drastic temperature fluctuations. These organisms must effectively sense and predict environmental changes, particularly the onset of desiccation. This review explores recent advancements in the molecular mechanisms that enable *N. flagelliforme* to survive under such harsh conditions, with a focus on stress signal sensing, transduction pathways, and photosynthetic adjustments. Key molecular adaptations include the production of extracellular polysaccharide (EPS) sheaths for water retention, the accumulation of compatible solutes like trehalose, and the synthesis of UV-absorbing compounds such as scytonemin and mycosporine-like amino acids (MAAs). Furthermore, *N. flagelliforme* utilizes a complex signal transduction network, including light-sensing pathways, to regulate responses to rehydration and desiccation cycles. This review emphasizes the integrative nature of *N. flagelliforme*’s adaptive mechanisms and highlights their potential for biotechnological applications, such as enhancing drought tolerance in crops and advancing ecological restoration in arid regions.

## 1. Introduction

Biological soil crusts (BSCs) cover 35% of the continents and exceed 70% of the living cover in parts of arid and semiarid region, playing a vital ecological role in enhancing soil stability and mitigating erosion, while simultaneously subjecting their microbial inhabitants to extreme desiccation, high irradiance, temperature fluctuations, and nutrient scarcity [1]. *Nostoc flagelliforme*, a filamentous cyanobacterium characterized by its distinctive black, hair-like colony morphology, is a dominant photosynthetic component of BSCs in the arid steppes of northwestern China and other semi-arid regions [2]. In its native habitat, *N. flagelliforme* spends much of its life in an air-dried quiescent state, reactivating metabolic activity intermittently when brief rain or nocturnal dew provides moisture [3]. Remarkably, *N. flagelliforme* exhibits the ability to endure repeated cycles of severe desiccation and rehydration, tolerating prolonged periods of near-complete dryness and resuming metabolic activity upon rehydration [4]. This finely tuned adaptation to “too dry to die, too wet to thrive” conditions reflects a highly specialized trait unique among cyanobacteria.

**Figure 1:**
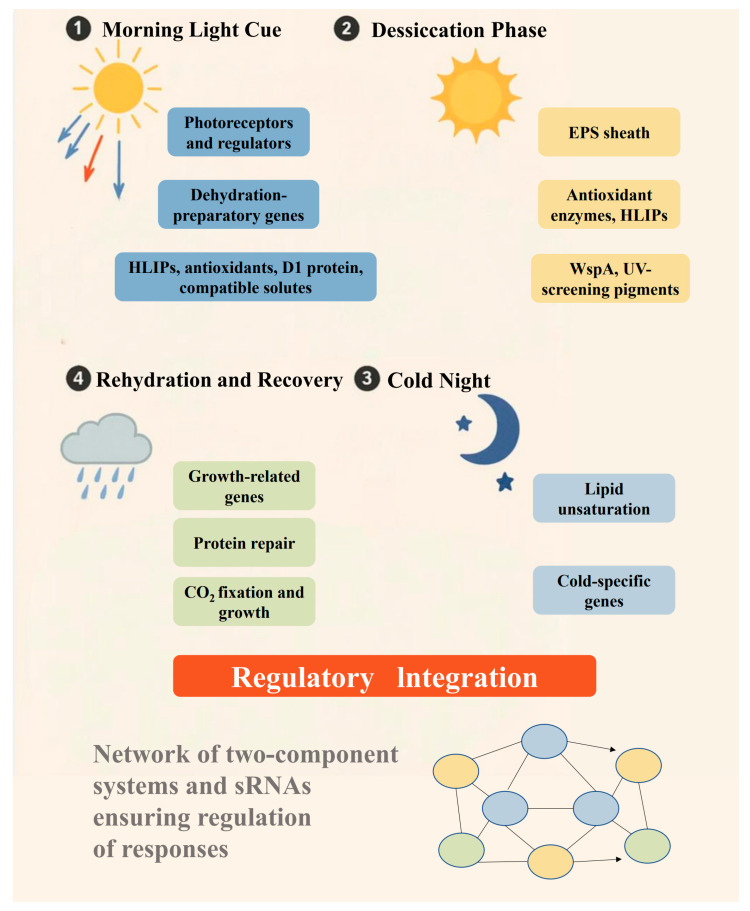
**Schematic of environmental adaptation cycle of *N. flagelliforme* in desert soil crusts**. *N. flagelliforme* experiences a daily cycle of hydration and desiccation in habitats, driven by dew at night and intense dryness during the day. (1) **Morning light cue**: At dawn, increasing blue and red light activates photoreceptors (e.g., NfPixJ, cryptochrome) and regulators (e.g., NfSrr1), triggering the expression of dehydration-preparatory genes. These include high-light inducible proteins (HLIPs), antioxidants, D1 protein, and compatible solutes to protect the organism from imminent desiccation. (2) **Desiccation phase**: As sunlight intensifies, *N. flagelliforme* loses water and enters dormancy. Its extracellular polysaccharide (EPS) sheath swells with the remaining moisture and then gradually dries, creating a buffered microenvironment. UV-screening pigments (scytonemin in the sheath and mycosporine-like amino acids in cells) absorb harmful radiation. Inside the cells, reactive oxygen species (ROS) are quenched by carotenoids and antioxidant enzymes, while HLIPs bind to chlorophyll, preventing photodamage. Water stress proteins (WspA, nowadays known to be a beta-galactosidase enzyme) and late embryogenesis abundant (LEA) proteins stabilize cellular structures, and photosynthesis is largely shut down, although photosystems remain intact, protected by chaperone proteins. (3) **Cold night**: As temperatures drop, *N. flagelliforme* adjusts by increasing lipid unsaturation and expressing cold-specific genes, such as *csrnf1*. In its desiccated state, the organism tolerates sub-freezing temperatures, preventing intracellular ice formation. (4) **Rehydration and recovery**: Upon receiving dew or rain at dawn, *N. flagelliforme* rapidly absorbs water. This triggers recovery pathways, possibly through the reversal of light signal pathways. Hrf1-mediated repression is lifted, allowing the expression of growth-related genes, including those for ribosomes and metabolism. Molecular chaperones (NfDnaK2/NfDnaJ9) and NfFtsH2 protease facilitate the repair of photosystem II (PSII) by replacing the damaged D1 protein, and phycobilisomes reattach as OCP-mediated quenching subsides. *N. flagelliforme* then begins CO_2_ fixation and growth until the next period of water exhaustion. **Regulatory integration**: Throughout the cycle, a network of two-component systems and small RNAs (sRNAs) modulate gene expression. This ensures that growth-related genes are repressed during the desiccation phase and activated during rehydration. This daily “boot-up and shutdown” cycle allows *N. flagelliforme* to persist through years of drought, highlighting the organism’s capacity for long-term survival in harsh environments.

The extreme environment of *N. flagelliforme* is characterized by multiple concurrent stresses. Daytime relative humidity in its habitat often remains below 60%, accompanied by intense sunlight and high evaporation, while nighttime brings cooler temperatures and higher humidity [2]. In its natural habitat, daily dew or sporadic rainfall initiates rapid water uptake, followed by equally rapid desiccation as the sun and dry air return [2]. *N. flagelliforme* has effectively adapted to “dry without dying”, surviving in a desiccated, dormant state and reviving its vegetative cells upon hydration events [5]. Solar radiation, particularly ultraviolet (UV) rays in these high-elevation steppes, imposes risks of DNA damage and oxidative stress [6]. Temperature fluctuations are significant, and in winter, the organism may experience freezing conditions [7]. Nutrient availability, especially combined nitrogen, is low in these dryland ecosystems [8]. To thrive under these combined stresses, *N. flagelliforme* has evolved a suite of interrelated morphological, physiological, and molecular adaptations that confer one of the most robust stress tolerance phenotypes known in photosynthetic organisms (Figure 1).

Research on *N. flagelliforme* dates back to the 1980s and 1990s when Chinese scientists documented its basic ecology, chemical composition, and potential for cultivation [2]. Early studies highlighted the cyanobacterium’s drought-hardiness and heat resistance, noting that metabolically active growth occurs during brief wet intervals (using dew or rain), and that colonies remain viable even after years of air-dried storage. The ability of *N. flagelliforme* to “dry without dying” in vegetative form (rather than producing specialized spores) led to comparisons with anhydrobiotic “resurrection plants” and other desiccation-tolerant life forms [5]. Concurrently, researchers observed vulnerabilities, such as the loss of structural integrity and invasion by decomposers under continuous hydration, indicating that *N. flagelliforme*’s biology is optimized for intermittently wet conditions [4]. These findings suggested that *N. flagelliforme* actively requires dry-down periods for longevity, supporting the idea that desiccation tolerance is an active, not passive, process.

With the advent of molecular tools, the past two decades have seen significant advances in uncovering the molecular genetics, phylogenetic characterization, and biochemical bases of *N. flagelliforme*’s environmental adaptations [9]. Notably, the complete genome of *N. flagelliforme* was sequenced, revealing a remarkably large genome (~10.2 Mb, among the largest in cyanobacteria) with extensive gene expansions in stress-related functions [10]. High-throughput transcriptomic studies, both in the field and laboratory, have illuminated gene expression changes during dehydration–rehydration cycles [10] and low temperature [7]. In addition to transcriptomic studies, proteomics [11,12] and metabolomics [13] have been carried out in *N. flagelliforme*. Parallel biochemical investigations have isolated novel protective compounds and characterized stress-responsive proteins unique to this organism [5,14]. These efforts have transformed *N. flagelliforme* into a model for understanding how cyanobacteria can survive extreme water limitations and radiation in terrestrial environments.

This review synthesizes current knowledge of the molecular mechanisms underlying *N. flagelliforme*’s environmental adaptation (Figure 1). Understanding these mechanisms is not only of fundamental scientific interest but also holds practical promise. Insights into its stress tolerance can inform ecological restoration efforts in arid regions. Moreover, the genes and pathways that confer extremotolerance in *Nostoc* may be transferred or engineered into crop plants or other organisms to enhance drought resistance and UV protection. In this review, we comprehensively examine the literature on *N. flagelliforme*’s adaptations, focusing on molecular and genetic mechanisms. We discuss these adaptations in thematic sections: Drought Adaptation Mechanisms; Ultraviolet Sunscreen Biosynthesis and Regulation; Photoprotection Mechanisms; Light Signal Transduction; and Cold Stress Adaptation. In each section, we outline key findings from recent studies and highlight remaining challenges. We conclude with a discussion of future directions and potential applications of this growing body of knowledge.

## 2. Drought Adaptation Mechanisms

Desiccation tolerance is central to *N. flagelliforme*’s survival strategy. This cyanobacterium inhabits environments where water is the most limiting resource, receiving moisture only from brief rain showers, dew, or fog, and enduring intense desiccation between these events [3]. *N. flagelliforme* has evolved a multifaceted array of mechanisms to cope with such extreme water stress.

### 2.1. Morphological and Life Cycle Strategies

**Figure 2:**
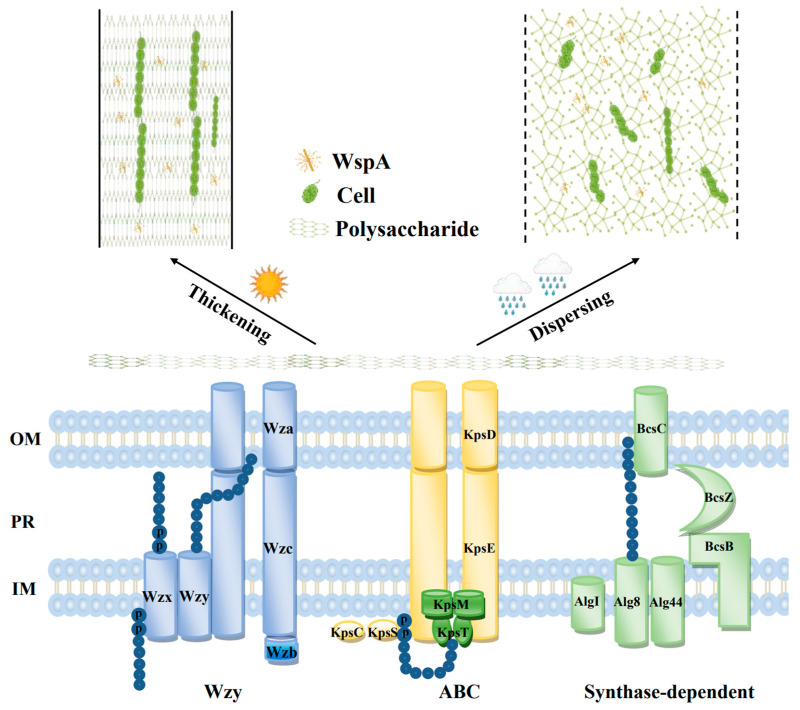
Schematic representation of the EPS assembly and export pathways (Wzy, ABC transporter, and synthase-dependent) and the WspA-facilitated flexibility and rigidity coordination of the EPS matrix in *N. flagelliforme*. OM, outer membrane; PR, periplasm; IM, inner membrane.

*N. flagelliforme* exhibits morphological and structural traits that confer advantages under drought conditions. It typically forms cylindrical, rope-like colonies composed of bundled filaments ensheathed in a thick extracellular polysaccharide (EPS) matrix [15]. A defining feature of *Nostoc* species is the production of a thick EPS sheath [16]. In *N. flagelliforme*, the EPS forms a matrix encasing many filaments, creating a rope-like colony. This sheath serves several protective functions: it binds and retains water, thereby slowing the rate of desiccation and providing a moist microenvironment during brief dry periods [4]. The polysaccharides of *N. flagelliforme* are primarily non-sulfated β-glycans, which can be secreted into surrounding soil, helping to cement soil particles into a crust when wet [4]. Thus, the EPS functions as both a biological sponge and shield, crucial for surviving during periodic desiccation–rehydration cycles (Figure 2). It is noteworthy that *N. flagelliforme*’s adaptation includes a rapid dehydration rate, and its fine filamentous colonies dry faster than the gelatinous colonies of *N. commune*, which may be beneficial [17]. Rapid water loss minimizes time spent in a stressful intermediate state, avoiding prolonged dampness that could invite microbial competitors or pathogens [2]. This trait, coupled with the water-holding EPS, suggests that *Nostoc* optimizes both water retention at the microscopic scale and water removal at the colony scale to thrive in its niche. Additionally, the filamentous colonies of *N. flagelliforme* form a mat structure that likely creates internal microgradients of humidity. During rehydration, the colony rapidly absorbs water, yet loses it quickly when the environment dries, ensuring that cells are not waterlogged for extended periods. *N. flagelliforme* cannot survive under continuously wet conditions because it becomes prone to decay or competition [4]. This dynamic water handling is thought to be a key aspect of its drought adaptation. In summary, the organism’s morphology (filamentous mats) and extracellular investments (thick sheath and EPS) are tailored to capture water quickly and release it swiftly, a strategy that aligns with the ephemeral water availability in desert environments. A remaining challenge is quantifying how these structural features contribute to water retention kinetics and determining the genetic basis for sheath composition differences. Future studies using mutants with altered EPS production could elucidate structure–function relationships in drought tolerance.

### 2.2. Cellular Osmoprotectants

When water becomes scarce, cells face osmotic stress and the risk of losing turgor pressure and dehydrating internally. *N. flagelliforme* mitigates this by accumulating compatible solutes, small organic molecules that protect cellular structures. Two key enzymes, maltooligosyl trehalose synthase (MTS) and trehalohydrolase (MTH), show significantly increased expression under osmotic, salt, and high-temperature stress [18]. Supplementing *Nostoc* cells with trehalose has been shown to preserve membrane integrity during desiccation [17]. In *N. flagelliforme*, high intracellular sucrose/trehalose levels correlate with improved drought recovery [19]. Trehalose stabilizes proteins and membranes by replacing water, preventing harmful aggregation or denaturation during drying in *Saccharomyces cerevisiae* [20]. It likely plays a similar role in *N. flagelliforme*, as suggested by analogies with other cyanobacteria: for example, desiccation-tolerant *Anabaena* strains also require trehalose for survival [21]. These sugars act as molecular protectants, preserving membrane phase integrity and enzyme structure until rehydration. In addition to organic protectants, maintaining ionic balance is another aspect of osmotic adaptation. *N. flagelliforme* likely extrudes sodium and accumulates potassium during dehydration to mitigate salt stress and maintain proper enzyme function [22]. These compatible solute strategies highlight cross-adaptation between drought and other stresses: the same molecules that protect against desiccation also confer tolerance to osmotic and salt stress, and even high temperature [21,23,24]. This multi-stress protection is advantageous in deserts, where drought often coincides with high salinity and heat. A challenge moving forward is identifying regulatory pathways that control osmolyte accumulation in *N. flagelliforme*. For example, what signals upregulate trehalose biosynthesis under stress (besides the direct effect of water loss)? Future research could explore the roles of global regulators (such as the salt-sensing two-component systems known in other cyanobacteria) or transcription factors that orchestrate the switch to osmoprotective metabolism during drying.

### 2.3. Desiccation-Induced Proteins and Protection Mechanisms

Many organisms that survive extreme drying produce specific proteins to protect macromolecules. For instance, late embryogenesis abundant (LEA) proteins and small heat shock proteins (sHsps) are common desiccation protectants in plants and some microbes [25,26]. *N. flagelliforme* has an expanded family of high-light inducible proteins (HLIPs), which are small chlorophyll-binding proteins that protect the photosystems during stress [5]. The complete genome sequence revealed an overrepresentation of genes for DNA replication, recombination, and repair [10]. This suggests that *N. flagelliforme* invests in maintaining genome integrity, which is crucial since desiccation can cause DNA damage. Extra copies of repair genes (such as photolyases, helicases, *recA*, etc.) facilitate efficient repair of DNA lesions upon rehydration [10]. Additionally, the genome encodes numerous antioxidant enzymes (superoxide dismutases and peroxidases), which prevent and mitigate damage during drought [27]. The metabolic shutdown that occurs during desiccation is accompanied by the expression of protective proteins to preserve cell viability in a suspended state. In addition to enzymatic antioxidants, *N. flagelliforme* secretes protective proteins into its extracellular sheath. One such protein is the water stress protein A (WspA), originally described in the desert cyanobacterium *Nostoc commune* [28]. *N. flagelliforme* contains two copies of the *wspA* gene, and homologous proteins are secreted into the EPS matrix and identified as beta-galactosidase enzyme [29]. Liu et al. [30] found that WspA and SOD were abundant in the secreted sheath of *N. flagelliforme*, where they coordinate the structural dynamics of the matrix during drying. Interestingly, *wspA* expression is upregulated under desiccation or UV stress in *N. commune* [28], and in *N. flagelliforme*, the genes are highly expressed in dry field samples (downregulated upon full rehydration). WspA appears to be a relatively rare feature, found in only a limited set of *Nostoc* species that experience extreme drying. This specificity underscores WspA’s role as a specialized adaptation to xeric habitats. Finally, *N. flagelliforme*’s cell membranes adapt to stress through lipid composition changes. A high proportion of monounsaturated and polyunsaturated fatty acids in membrane lipids has been documented in *N. flagelliforme* [31], which is advantageous for maintaining membrane fluidity and phase stability under both drying and low-temperature stress [31]. Unsaturated lipids prevent membranes from becoming too rigid when desiccated or too solid when cold, thereby protecting membrane protein function [32]. This trait exemplifies a biochemical overlap between drought and other stresses (e.g., heat and cold), reinforcing the concept of cross-adaptation.

### 2.4. Challenges and Future Directions in Drought Adaptation Research

Studying *N. flagelliforme*’s drought tolerance presents several challenges, particularly due to the difficulty in cultivating this organism under laboratory conditions. However, with the sequencing of its 10.23 Mb genome and the successful application of CRISPR-based mutagenesis, significant progress is being made, providing the opportunity to experimentally verify the function of drought-related genes. Future research should focus on the construction of mutants or overexpression strains to investigate the role of genes involved in extracellular polysaccharide (EPS) biosynthesis, trehalose synthesis, antioxidant defense, and secretion systems in desiccation survival. Another promising direction is addressing the “too much water” paradox: understanding the molecular mechanisms that trigger sheath disintegration during prolonged hydration and the ecological significance of this process. This may require examining the interactions between *N. flagelliforme* and its symbiotic bacteria residing in the EPS sheath. Lastly, comparative genomics between xerotolerant and non-xerotolerant *Nostoc* species could help identify gene sets unique to xerotolerance, such as the overrepresentation of transposases and DNA repair genes in *N. flagelliforme*. Investigating how these genes contribute to genomic plasticity and adaptation to dryland ecosystems will illuminate the evolutionary strategies—perhaps through promoting rapid genome rearrangements or facilitating horizontal gene transfer of stress-related genes—which presents an exciting avenue for future research.

In summary, *N. flagelliforme*’s drought adaptation is a concerted effort involving physical, chemical, and biological strategies. Its EPS sheath and colony form manage water relations externally or internally, and it accumulates osmoprotective solutes and expresses protective molecules to safeguard proteins, membranes, and DNA. The organism’s genome reflects these strategies, with expanded gene families for stress tolerance and repair [10]. These mechanisms together enable *N. flagelliforme* to endure the paradox of life on the edge of water availability: it survives desiccation but not death, ready to spring back to life when the desert rains arrive.

## 3. Ultraviolet Sunscreen Biosynthesis and Regulation

*N. flagelliforme*, inhabiting high-elevation desert habitats, is exposed to intense solar radiation, including harmful ultraviolet (UV) A (320–400 nm) and B (280–320 nm) wavelengths. Desiccated or slow-growing cyanobacterial cells are particularly vulnerable to UV-induced DNA damage and oxidative stress [10]. To counteract these challenges, *N. flagelliforme* has evolved robust photoprotective sunscreens that absorb UV radiation, preventing cellular damage [9]. Field observations indicate remarkable UV tolerance in *N. flagelliforme* colonies. For instance, Gao and Ye [33] found that the photosynthetic activity of *N. flagelliforme* remained essentially unaffected by ambient solar UV, both in dried and hydrated states. The cyanobacterium achieves this resilience through the production of natural sunscreens that absorb UV radiation, preventing its penetration into vital cellular components. Two primary types of cyanobacterial sunscreens are scytonemin and mycosporine-like amino acids (MAAs).

### 3.1. Scytonemin

Scytonemin, a lipid-soluble indole-alkaloid pigment, is found in the extracellular sheath of many desert cyanobacteria, where it serves as a UV-A sunscreen [34]. The dark brownish-black coloration of *N. flagelliforme*’s sheath is partially due to the deposition of scytonemin. Ferroni et al. [35] demonstrated that *N. flagelliforme* produces scytonemin in conjunction with MAAs, and these two sunscreens have complementary absorption profiles, covering UV-A and UV-B, respectively. Scytonemin biosynthesis is induced by UV-A light, and genes involved in its production (the *scy* cluster) are upregulated upon exposure in related *Nostoc* species [36,37]. This explains why *N. flagelliforme* can remain desiccated on the soil surface under intense sunlight, later reviving when moisture becomes available. However, the molecular basis of scytonemin biosynthesis regulation remains unexplored. It remains unclear whether the putative two-component regulator upstream of the *scy* cluster is responsible for transcriptional regulation. Further investigation is required to determine how this regulator relates to environmental sensory processes, signal transduction, gene expression, and posttranscriptional regulation.

### 3.2. Mycosporine-like Amino Acids (MAAs)

MAAs are small, water-soluble compounds that absorb UV radiation, primarily UV-B and short UVA, with high molar absorptivity. Recent studies have revealed that *N. flagelliforme* synthesizes a unique MAA [38]. Shang et al. [9] identified a five-gene cluster in *N. flagelliforme* responsible for MAA biosynthesis. Genetic and chemical analyses identified the compound as mycosporine-2-(4-deoxygadusolyl-ornithine), an unusual MAA containing an ornithine side group (molecular weight 756 Da). The absorption spectrum of this compound features a distinctive double peak, which broadens its protective range, making it the largest and most structurally unique MAA reported to date [9]. This discovery highlights *N. flagelliforme*’s sophisticated UV defense mechanisms and expands the known diversity of MAAs in nature. While most cyanobacterial MAAs, such as shinorine and porphyra-334, feature serine or glycine attachments, the ornithine-containing MAA in *N. flagelliforme* suggests a novel adaptation. Ornithine, a basic amino acid, may enhance the stability or UV absorbance of the molecule. Genome analysis revealed that *N. flagelliforme* possesses an “ornithine-ammonia cycle” that enables efficient production of ornithine from arginine via a specialized arginine dihydrolase (ArgZ). This pathway, elucidated by Zhang et al. [39], connects nitrogen storage (through cyanophycin degradation) to MAA synthesis. During stress, *N. flagelliforme* degrades cyanophycin, a polymer of arginine and aspartate, to release arginine. This is then converted to ornithine and ammonia (via ArgZ) and channeled into MAA biosynthesis. The evolution of an ornithine-based MAA may reflect positive selection for improved UV protection in the organism’s harsh environment. Phylogenetic analyses suggest that this MAA pathway could be unique to subaerial cyanobacteria [10]. The MAA biosynthesis pathway was successfully transferred to *Nostoc* sp. PCC 7120 in the laboratory, which then produced the same MAA [9].

### 3.3. Regulation of MAA Synthesis

The regulation of MAA biosynthesis in *N. flagelliforme* marks a significant advancement in understanding UV responses. Shang et al. [9] identified a regulatory protein, OrrA, which acts as a positive UV-B-responsive regulator of the MAA gene cluster. OrrA binds to the promoter of the *mys* genes, activating their transcription upon UV-B exposure. Overexpressing *orrA* enhanced UV tolerance in *N. flagelliforme* during desiccation and facilitated photosynthetic recovery upon rehydration [9]. The name OrrA (osmo- and radiation-response regulator A) reflects its dual role. It was initially associated with osmotic stress in *Anabaena*, where it controls sucrose synthesis in response to salt stress [24]. In *N. flagelliforme*, OrrA appears to sense UV light, inducing both sunscreen production and other stress responses. Notably, OrrA contains an N-terminal receiver domain and a C-terminal helix-turn-helix domain, typical of two-component response regulators, suggesting that it may receive a phosphorylation signal from an unknown sensor upon UV-B exposure. Thus, the regulation of sunscreen production in *N. flagelliforme* is integrated into a classic two-component signaling pathway: UV-B signals activate OrrA, which in turn triggers the expression of enzymes involved in MAA sunscreen synthesis. Identifying its upstream sensor remains an open question. OrrA represents a clear example of transcriptional control over protective metabolites in *N. flagelliforme*.

### 3.4. Future Directions

Characterizing other transcriptional regulators involved in UV responses will offer a deeper understanding of UV protection mechanisms. Are there sigma factors or small RNAs modulating the expression of UV-protection genes? A recent study discovered a small RNA (PsrR1) in *Synechocystis* sp. PCC 6803 [40]. In addition to regulating photosynthesis in unicellular cyanobacteria, PsrR1 may regulate photosynthesis during rehydration in *N. flagelliforme* [10]. Beyond PsrR1/SyR1, which modulates photosystem I gene expression under high-light stress to maintain a balanced photosynthetic electron flow [40], several nitrogen-responsive sRNAs have been characterized in the model filamentous cyanobacterium *Nostoc* sp. PCC 7120. Among these, the tandemly repeated NsiR1 cluster is rapidly induced during early heterocyst differentiation and post-transcriptionally represses the expression of *hetF*, fine-tuning the commitment to specialized cell-type development [41]. Under sustained nitrogen starvation, the sRNA NsrR1 coordinates with the global nitrogen regulator NtcA to downregulate *nblA*, thus preventing premature phycobilisome degradation [42]. More recently, NsiR4 has been demonstrated to integrate carbon and nitrogen metabolic pathways by targeting key Calvin cycle enzymes, thereby reducing CO_2_ fixation under nitrogen-limited conditions [43]. Collectively, these examples highlight the diverse repertoire of sRNAs employed by Nostocales in orchestrating photosynthesis, nutrient assimilation, and cell differentiation, emphasizing the necessity of incorporating these sRNA-mediated regulatory circuits into comprehensive models of environmental acclimation in *N. flagelliforme* and related cyanobacterial taxa.

Perhaps analogous sRNAs exist for UV stress. Does UV induce unique responses beyond general stress? How do multiple signals, such as light quality, hydration state, and temperature, combine in *N. flagelliforme*? Are there dedicated UVA and UVB photoreceptors? If so, how are these signals transduced? Addressing these questions is critical for understanding how organisms cope with concurrent environmental stresses.

In summary, *N. flagelliforme* combats UV stress through a two-pronged sunscreen strategy: scytonemin in the sheath provides broad, constitutive UV-A protection, while inducible MAAs offer dynamic UV-B/A protection. The transcriptional regulation of these pathways (via regulators like OrrA) exemplifies how this cyanobacterium senses harmful radiation and proactively boosts its defenses. These insights not only advance our understanding of cyanobacterial photobiology but also hold biotechnological potential. For example, the unique MAA could be harvested or synthesized as a high-value sunscreen for human use, and the *orrA* gene might be used to engineer UV-responsive protective systems in other microorganisms.

## 4. Photoprotection Mechanisms: OCPs, HLIPs, and Molecular Chaperones

High solar irradiance in desert habitats presents a dual threat to photosynthetic organisms: not only does it expose them to harmful UV radiation, but intense visible light can overwhelm the photosynthetic apparatus, leading to photoinhibition [44] or oxidative damage [45]. *N. flagelliforme* must manage excess sunlight absorption, particularly during desiccation or when metabolic activity is slowed, as normal photochemistry is impeded and excess light can generate reactive oxygen species (ROS). Photoprotection refers to the suite of mechanisms that safeguard the photosystems (PSI and PSII) and other cellular components from damage caused by excess light and ROS. In *N. flagelliforme*, two well-known photoprotective protein families—high-light inducible proteins (HLIPs) [5] and orange carotenoid proteins (OCPs) [14]—are significantly expanded compared to typical cyanobacteria. The expansion of these gene families in *N. flagelliforme* highlights their critical role in the organism’s adaptation to its extreme environment [10].

### 4.1. High-Light Inducible Proteins (HLIPs)

HLIPs are small membrane proteins (typically ~30 amino acids) containing a single chlorophyll-binding helix, which is related to light-harvesting antenna proteins [46,47]. These proteins are rapidly induced under high light or other stress conditions and are believed to bind chlorophyll or other pigments, thereby dissipating excess excitation and preventing the formation of harmful free chlorophyll that can generate singlet oxygen [46,47]. *N. flagelliforme* contains 12 distinct *hlip* genes (more than double the cyanobacterial average of approximately five), along with a unique cluster of four tandemly duplicated *hlip* genes [10]. Xu et al. [5] discovered that these four HLIPs, forming an HLIP cluster, are strongly induced by dehydration in *N. flagelliforme*. The expansion of this gene family likely arose from gene duplication. Phylogenetic analysis suggests that most *Nostoc* species have some degree of HLIP amplification, coupled with a specific regulatory gene.

Using CRISPR-based gene editing, Xu et al. [5] created a mutant of *N. flagelliforme*, where the entire *hlip* cluster was deleted, and functionally validated its critical role. The mutant exhibited significantly reduced desiccation tolerance and impaired PSII recovery upon rehydration, suggesting that HLIPs are required to protect and/or repair PSII during the drying process. Furthermore, the expression of *N. flagelliforme*’s *hlip* cluster in *Nostoc* sp. PCC 7120 enhanced desiccation tolerance in the latter, reinforcing the notion that HLIPs confer a transferable protective benefit.

Interestingly, Xu et al. [5] identified a transcription factor, Hrf1 (HLIP-cluster repressor factor 1), that directly regulates the *hlip* cluster. Hrf1 is an RpaB-like regulator, with RpaB in other cyanobacteria being a redox-sensitive transcription factor that controls many light-responsive genes [48]. Hrf1 binds the promoter of the *hlip* cluster and represses it under normal conditions. In an *hrf1* knockout mutant, the *hlip* cluster was de-repressed and exhibited even higher expression upon dehydration than in the wild type. Moreover, Hrf1 regulates certain *psbA* genes, which encode D1 variants of PSII that are induced by dehydration. This coordination suggests that Hrf1 synchronizes the expression of photoprotective proteins (HLIPs) with PSII repair enzymes (like D1 and associated proteases) during desiccation. The model proposed by Xu et al. [5] suggests that, under dehydration stress, Hrf1 activity is modulated (likely by redox changes or other stress signals), lifting the repression on the *hlip* operon and promoting the expression of *psbA* genes, thereby facilitating effective PSII repair once water becomes available. This regulatory adaptation demonstrates how a factor originally involved in high-light response (RpaB homolog) has been co-opted to manage desiccation-induced photoprotection. The coevolution of the expanded *hlip* cluster and *hrf1* in *Nostoc* species underscores their concerted role in the evolution of desiccation tolerance, a trait that likely emerged in an ancestor and was pivotal for the colonization of terrestrial habitats.

### 4.2. Orange Carotenoid Proteins (OCPs)

Cyanobacteria possess a unique photoprotective mechanism known as non-photochemical quenching (NPQ) of phycobilisome fluorescence, mediated by the orange carotenoid protein (OCP) [49]. OCP is a soluble protein that binds a carotenoid, and under strong blue-green light, OCP undergoes photoactivation, causing it to attach to the phycobilisome and quench its fluorescence, thereby dissipating excess excitation energy as heat [50]. This prevents over-excitation of the reaction centers and the generation of harmful ROS. Most cyanobacteria have a single OCP, along with a helper protein (FRP) that resets OCP activity [51]. Remarkably, subaerial cyanobacteria such as *N. flagelliforme* possess an expanded repertoire of OCP-related proteins: two full-length OCPs (OCPx1 and OCPx2), four helical carotenoid proteins (HCPs; HCP1, 2, 3, and 6), and one C-terminal domain-only protein (CCP) [10,52]. This diverse suite of proteins reflects functional specialization.

The four HCPs (helical carotenoid proteins, lacking the OCP effector domain) are excellent singlet oxygen quenchers, with HCP2 being the most efficient [14]. This suggests that HCPs primarily function as antioxidants, neutralizing ROS. In contrast, the two full OCPs (OCPx1 and OCPx2) do not quench singlet oxygen efficiently but perform photobleaching of phycobilisome fluorescence, effectively regulating energy flow in the antenna. Structural analysis revealed that OCPx1 is fast and strongly quenching, whereas OCPx2 is slower and has unique properties compared to known OCPs. OCPx2 tends to remain monomeric and flexible, whereas OCPx1 forms oligomers, suggesting different regulatory or interaction properties [14].

The combination of multiple OCP/HCP proteins allows *N. flagelliforme* to address both primary oxygen radicals and excess excitation energy. This represents an adaptive evolution for photoprotection: HCPs protect the cell by directly quenching ROS, while OCP variants protect by dissipating excess absorbed energy at the phycobilisome. The presence of these paralogs in subaerial *Nostoc* species indicates strong selective pressure in terrestrial high-light environments to diversify photoprotective strategies.

### 4.3. Molecular Chaperones

Photoprotection also involves the efficient repair of damaged components. During dehydration, even with quenching systems in place, some damage to PSII (especially the D1 protein) is inevitable due to high light and limited electron transport. *N. flagelliforme* has evolved a specialized molecular chaperone system to aid PSII repair (most notably the NfDnaK2 chaperone and its co-chaperone NfDnaJ9) [53]. DnaK is an Hsp70-family chaperone, and cyanobacteria often have multiple isoforms [54]. In *N. flagelliforme*, NfDnaK2 is strongly induced by dehydration and predominantly localizes to the thylakoid membranes [53]. Alongside NfDnaK2, NfDnaJ9 is co-induced by dehydration and physically interacts with NfDnaK2. The NfDnaK2/NfDnaJ9 pair associates with the NfFtsH2 protease, which degrades the damaged D1 protein in PSII [53].

Xu et al. [53] showed that heterologous expression of NfDnaK2 in *Nostoc* sp. PCC 7120 significantly improved the strain’s drought tolerance and PSII repair rate. Essentially, NfDnaK2 enhances the efficiency of D1 turnover by stabilizing intermediate complexes or preventing the aggregation of partially disassembled PSII, thereby maintaining the integrity of the photosynthetic machinery during stress and allowing rapid recovery upon rehydration. The same study identified two transcriptional regulators, NfRre1 and NfPedR, that bind the promoter of NfDnaK2 and likely mediate its induction in response to dehydration. NfRre1 is a response regulator (NarL/FixJ family), possibly linked to nitrogen or osmotic signaling, while NfPedR is a regulator related to photosynthetic electron transport, potentially sensing redox changes. These regulators illustrate how *N. flagelliforme* integrates environmental signals to activate a protective chaperone system when needed.

### 4.4. Other Photoprotective Factors

In addition to HLIPs, OCPs, and the NfDnaK2 system, *N. flagelliforme* likely employs other photoprotective mechanisms. Its cells produce various carotenoids (e.g., echinenone and myxoxanthophyll) in their membranes, which quench triplet chlorophyll and free radicals [55]. The strong desiccation tolerance of *N. flagelliforme* has been partly attributed to a high carotenoid-to-chlorophyll ratio in the dried state, as evidenced by its intensified orange-brown coloration when dry, indicating a higher carotenoid concentration. Enzymatic antioxidants, such as superoxide dismutase (SOD) and ascorbate peroxidase in the thylakoids, help detoxify superoxide or peroxides formed under high light [11,56]. Additionally, flavodiiron proteins (FLVs), expanded in some *Nostoc* species, serve as electron sinks to relieve excess electrons from PSI, thereby reducing photoreduction of oxygen (which forms ROS) [57,58]. The presence of multiple copies of certain global regulators, such as sigma factors and two-component systems, also suggests the organism’s ability to swiftly adjust the composition of its photosynthetic apparatus in response to light changes.

These photoprotective strategies collectively ensure that *N. flagelliforme* can endure intense desert sunlight without suffering irreversible photodamage. By dissipating excess energy as heat (NPQ via OCPs), binding free chlorophyll (HLIPs), scavenging ROS (HCPs, carotenoids, enzymes), and dynamically remodeling the photosynthetic apparatus, *N. flagelliforme* avoids the “phototoxicity of free chlorophyll” and other light-induced lesions even under extreme conditions. These mechanisms are critical upon rehydration, when a sudden influx of water allows full photosynthesis to resume, often under very bright morning light. Future research should investigate the cross-talk between light signaling and photoprotection. For instance, does the red-light NfSrr1 [59] pathway contribute to preconditioning photoprotective responses? Another promising avenue is the potential application of *N. flagelliforme*’s photoprotective genes in other systems. Specifically, could the introduction of the *hlip* cluster or *ocp* variants into crop plant chloroplasts enhance their tolerance to drought or high-light stress? While this synthetic biology approach presents challenges in terms of gene expression and targeting, the proven effectiveness of these proteins in cyanobacteria suggests that such strategies could offer significant benefits.

## 5. Light Signal Transduction

One of the most intriguing recent findings is that *N. flagelliforme* can “read” ambient light cues to anticipate and prepare for desiccation [3]. The daily light cycle in desert environments provides reliable cues: dawn and early morning light conditions signal imminent drying as the sun rises, while dusk light indicates the arrival of a moist, cool night [60]. *N. flagelliforme*, being diurnally active, has evolved photoreceptors and signaling pathways that transduce these environmental light changes into physiological adjustments, enhancing its desiccation tolerance [59].

### 5.1. Diurnal Light as an Anticipatory Signal

Cyanobacteria perceive and respond to light not only for photosynthesis but also as an informational signal regarding environmental conditions. For desert cyanobacteria, diurnal light changes serve as predictive cues for impending desiccation. Oren et al. [60] demonstrated that low-intensity illumination at dawn triggers a preparatory response in *Leptolyngbya ohadii*, a desert cyanobacteria found in BSCs, thereby enhancing desiccation tolerance. This early morning light cue activates protective genes before the moisture evaporates. Desert cyanobacteria typically experience a pattern where moisture from dew or brief rain is available in the early morning, followed by intense sunlight and heat, leading to drying by midday. Thus, early morning light, particularly low-angle red light, can indicate impending drying. The ability to “read” these cues and prepare accordingly enhances survival, a concept termed anticipatory stress response. Consistent with this, Gao et al. [2] showed that *N. flagelliforme*’s recovery of photosynthesis is light-dependent, suggesting that light triggers specific cellular processes essential for photosynthesis restoration. Xu et al. [61] further reported that weak red light, in particular, plays a crucial role in awakening the photosynthetic machinery following desiccation in *N. flagelliforme*. This suggests that *N. flagelliforme* might utilize the red/far-red light sensor phytochrome for signaling, as phytochromes typically respond to red light.

### 5.2. Red Light Sensing Pathway (NfPixJ–NfSrr1)

The mechanistic basis for this red-light effect has been traced to a specific signaling pathway involving a cyanobacteriochrome photoreceptor and a transcription factor. Xu et al. [59] demonstrated that red light serves as a key anticipatory signal, triggering a cascade mediated by the cyanobacteriochrome photoreceptor NfPixJ and the transcription factor NfSrr1 in *N. flagelliforme*. NfPixJ, a type of bilin-binding photoreceptor with multiple GAF domains, absorbs red/far-red light and undergoes photoconversion. Biochemical assays revealed that NfPixJ binds phycocyanobilin chromophores, exhibiting red/green photoreversible absorption, with one GAF domain specifically binding the chromophore. NfSrr1, a cytosolic transcription factor of the LuxR family, physically interacts with NfPixJ in a light-dependent manner, as demonstrated by yeast two-hybrid and pull-down assays. Under red-light exposure, NfPixJ likely undergoes a conformational change, activating or releasing NfSrr1, which then regulates target genes. Among the key targets are genes involved in photosystem II (PSII) maintenance, including multiple *psbA* genes encoding the D1 protein of PSII and *ftsH2*, a protease involved in D1 degradation. Red light during dehydration strongly induces these genes in an NfSrr1-dependent manner.

A striking target of NfSrr1 regulation is the *psbA* gene set encoding a D1 protein variant (Q130E), where a glutamine at position 130 is replaced by glutamate. This modification enhances PSII resistance to high light and oxidative stress [62,63]. NfSrr1 physically binds to the promoters of *psbA2*, *psbA5*, and *ftsH2*, enhancing their transcription. In an *Nfsrr1* knockout mutant, this induction is lost, and desiccation tolerance is significantly reduced. Thus, the NfPixJ–NfSrr1 pathway functions as a red-light-triggered switch that activates PSII repair and protection mechanisms before desiccation damage occurs. This pathway links the perception of red light to the activation of protective biochemical mechanisms, with the NfSrr1 pathway also regulating the synthesis of compatible solutes and other stress-related metabolites, beyond just photosystem components [59]. These findings suggest a broad role for red light signaling, preparing the cell by accumulating osmoprotectants and stabilizing proteins and membranes ahead of water loss.

### 5.3. Expanded Photoreceptor Network

While red light signaling through the NfPixJ–NfSrr1 pathway is a significant discovery, *N. flagelliforme* likely senses additional wavelengths via other photoreceptors, including cryptochromes/photolyases (UV-A/blue light sensors), LOV-domain proteins (blue light), and other cyanobacteriochromes that detect a range of colors (blue, green, red, and far-red) [3,64]. The interplay between these pathways is still being explored. Shang et al. [10] noted that at least two classical *cph1* genes encoding phytochromes are present in *N. flagelliforme*. Transcriptomic data show dynamic expression changes in the *cph1* gene upon rehydration, suggesting that the phytochrome signaling pathway is active during the early stages of the wet–dry transition, potentially regulating genes necessary for photosystem reactivation [59].

In addition to red light, blue light appears to be an important cue in the early morning. Desert dawn light is often rich in diffuse blue wavelengths before direct sunlight, which transitions to red as the sun rises. Experiments with the soil crust cyanobacterium *Leptolyngbya ohadii* showed that blocking blue light at dawn reduced desiccation recovery [60], highlighting the role of blue light signaling. Although the red light pathway in *N. flagelliforme* is clearly critical, other photoreceptors may modulate additional responses. Oren et al. [60,64] observed differential expression of over 1500 genes in another crust cyanobacterium in response to far-red versus white light during drying, including genes related to compatible solute turnover and protective complexes. This suggests that photoregulation of desiccation responses is widespread and involves multiple photoreceptors responding to different light wavelengths.

*N. flagelliforme* is well equipped in this regard, with its genome encoding at least two conventional phytochrome family sensors (*cph1* genes) and nine distinct cyanobacteriochrome photoreceptors containing GAF domains that respond to various colors [53]. In vitro assays confirmed that these cyanobacteriochromes from *N. flagelliforme* can detect different light wavelengths. The organism essentially has a “color vision” system, allowing it to fine-tune its responses depending on whether the ambient light is dominated by blue (indicating dawn or shade), red (full sunlight in the morning), or far-red (late day or filtered light) light. During a typical day, *N. flagelliforme* experiences a progression from moist and low light at night, to increasing blue and red light at dawn, intense midday sun, desiccation by afternoon, and potential rehydration at dusk. The sensing of dawn triggers preparation for desiccation, while the shift to far-red light signals the potential for rewetting as the day ends.

The daily cyclic nature of these light signals means that *N. flagelliforme*’s stress responses are rhythmic or anticipatory rather than purely reactive. A concrete example of this coupling is the far-red light effect: far-red light (~730 nm) can antagonize red light in phytochrome systems, where far-red light drives the photoreceptor to an inactive state. In *L. ohadii*, continuous far-red light during drying negated the positive effect of red light on desiccation tolerance, leading to poor recovery [60]. This is consistent with phytochrome-mediated sensing, where far-red light indicates conditions not conducive to desiccation. *N. flagelliforme* may interpret a dominance of far-red light as a cue that it is not in direct sunlight, avoiding the full dehydration-hardening response and focusing on maintenance instead. In contrast, the combination of rising blue and red light at dawn clearly signals that a drying period is imminent, triggering the full protective program.

### 5.4. Challenges and Future Directions

One significant challenge is differentiating between direct and indirect light effects. For example, red light may not only signal impending drought but also enhance photosynthesis during the early morning, which in turn alters the internal redox balance, potentially acting as a signaling mechanism itself. While the discovery of the NfPixJ–NfSrr1 pathway provides a valuable starting point, the intermediate steps, specifically, how NfPixJ activation leads to NfSrr1-dependent gene transcription, require further elucidation. Additionally, the interaction between light signaling and other environmental sensors, such as those for osmotic stress or temperature changes, remains an underexplored area. From an applied perspective, understanding how red light primes cells could lead to innovative strategies for preconditioning cyanobacterial cultures. For instance, determining whether brief exposure to red light could enhance the survival of cyanobacterial inoculants used in biocrust restoration prior to their deployment in harsh field conditions could have significant ecological implications.

In summary, *N. flagelliforme* has evolved a highly sophisticated light-sensing apparatus that aligns its stress responses with the daily rhythm of its environment. The discovery of the NfPixJ–NfSrr1 red light pathway exemplifies how organisms exploit natural correlations, such as morning light preceding afternoon drought, to their advantage. This finding not only enhances our understanding of *Nostoc* biology but also contributes to the broader field of stress signaling in cyanobacteria. It could inspire bioengineering strategies, such as designing crops that “know” drought is coming by sensing cues like light or temperature and pre-activating protective genes. Furthermore, the interplay between light signaling and other sensors, such as osmotic or temperature sensors, during dehydration remains an exciting area for future research.

## 6. Cold Stress Adaptation

Dryland habitats impose not only chronic water limitation and intense daytime heat but also striking diurnal and seasonal thermal oscillations. In the Inner Mongolian desert steppe, for example, surface soil temperatures can plunge well below 0 °C at night and throughout the winter, generating recurrent freeze–thaw cycles that challenge resident biota [65]. For the filamentous cyanobacterium *N. flagelliforme*, cold tolerance is therefore a critical complement to its well-studied drought hardiness. Like other cyanobacteria, it counters low-temperature stress by (i) remodeling membrane lipids through the up-regulation of fatty acid desaturases to preserve fluidity [66], (ii) synthesizing cold-shock proteins and RNA chaperones that stabilize transcription–translation machinery [67], and (iii) reconfiguring central metabolism to sustain redox and energy balance [68,69]. Recent transcriptomic profiling under both acute chilling and prolonged winter-like conditions revealed coordinated induction of genes for desaturation, compatible solute biosynthesis, antioxidant defenses, and protein quality control, underscoring the capacity of *N. flagelliforme* to withstand sudden cold shocks as well as extended dormancy during the frozen season [7].

### 6.1. Membrane Lipid Desaturation

One of the fastest and most universal responses to a temperature downshift in cyanobacteria is to increase the degree of unsaturation of fatty acids in membrane lipids [70]. This prevents membranes from becoming too rigid at low temperatures. As mentioned, *N. flagelliforme* inherently has a high proportion of monounsaturated and polyunsaturated fatty acids in its membranes [31]. Liu et al. [71] reported that in *N. flagelliforme* cultures, growth at lower temperatures led to elevated unsaturated fatty acid content in the cells, maintaining membrane fluidity. Genes encoding acyl-lipid desaturases (e.g., *desA* and *desB*) are present in its genome and likely up-regulated by cold. The comparative genomics study by Shang et al. [10] noted the acquisition of genes for fatty acid desaturation as part of *N. flagelliforme*’s genome expansion. Thus, when exposed to cold nights or seasonal cooling, *N. flagelliforme* ensures its cellular membranes remain semi-fluid, allowing necessary transport and enzyme functions to continue.

### 6.2. Cold-Responsive Hypothetical Protein—Csrnf1

A particularly novel discovery by Gao et al. [7] was the identification of a previously hypothetical gene, named *csrnf1* (cold stress resistant *Nostoc flagelliforme* gene 1), that is strongly responsive to cold. It appears to be single-copy and broadly conserved in many cyanobacterial genomes (266 homologs found, mainly in Nostocales and Chroococcales), though absent in non-cyanobacteria. To probe its function, Gao et al. [7] created a transgenic *Nostoc* sp. PCC 7120 strain expressing *csrnf1*. This *csrnf1*-overexpressing strain showed markedly improved growth and survival at a low temperature (15 °C) compared to the wild type, confirming that *csrnf1* enhances cold resistance. Intriguingly, the same transgenic strain was also more tolerant to nitrogen depletion stress. This hints that *csrnf1* might have a multi-functional role, aiding in stress resilience under both cold and nutrient limitation. The exact mechanism of *csrnf1*’s action remains unknown—its sequence lacks obvious motifs, and attempts to find domains did not yield clear results. It might be a unique regulatory protein or enzyme that, for example, modulates membrane composition or signaling pathways under stress. The fact that it clusters phylogenetically, somewhat by cyanobacterial lineage, suggests that it may have evolved divergent roles in different groups. In *N. flagelliforme*, it is clearly important for enduring cold, dry winters on infertile soils, where both temperature and nutrient availability (especially fixed nitrogen) can be low. Csrnf1 could represent a novel type of stress protein, and its discovery underscores the value of transcriptomic mining of extremophiles for previously uncharacterized genes.

### 6.3. Challenges and Future Directions

Studying *N. flagelliforme*’s cold survival poses several challenges. First, it is hard to replicate the exact desert conditions in the lab. Realistic diel cycles of temperature, humidity, and light are complex, and static stress assays cannot recreate the interplay of cold, dryness, and rewetting that occurs in nature. Second, there are no published proteomic or metabolomic profiles for *N. flagelliforme* under cold or night stress, so the downstream effects of gene changes are largely unknown. Third, ecosystem context is often overlooked. In nature, *N. flagelliforme* lives in a community. Bashir et al. [72] reported that it associates with diverse bacteria and even secretes over 200 enzymes and transport proteins during rehydration to interact with epiphytic microbes. Thus, laboratory studies on *N. flagelliforme* alone miss these ecological interactions, which could critically influence nutrient uptake and stress protection at night. Fourth, on the signaling side, the sensors and regulators of cold (e.g., two-component systems, alternative sigma factors, or circadian clock genes) remain unexplored in *N. flagelliforme*. These factors collectively represent key hurdles to fully understanding its night-time cold survival.

To overcome these challenges, integrated and interdisciplinary approaches are needed. Time-series omics studies are a priority: combining transcriptomics with proteomics and metabolomics under controlled diel cycles would reveal how gene expression translates to proteins and metabolites through a full night–day cycle. Metagenomic and metatranscriptomic surveys of biological soil crusts would illuminate community interactions: which microbial partners supply nutrients or signals to *N. flagelliforme* at night? Another promising direction is to dissect light and temperature signaling systems in *N. flagelliforme*. The observed red light response implicates a phytochrome-like photoreceptor; elucidating its identity and downstream signaling cascade will clarify how dawn cues are converted into metabolic activation [59]. Investigating circadian clock genes could reveal whether *N. flagelliforme* anticipates the night–day cycle on a molecular level. In parallel, biochemical and biophysical studies (e.g., characterizing membrane properties at low T) would deepen understanding of cellular adjustments. Overall, cold adaptation in *N. flagelliforme* complements its drought and light adaptations, ensuring year-round survival across seasonal extremes of the desert climate.

## 7. Conclusions and Future Perspectives

*N. flagelliforme* has emerged as a model organism for understanding how life can persist and thrive under extreme environmental stress. Throughout this review, we have demonstrated that its survival in arid, UV-exposed, and cold desert habitats is not the result of a single “magic bullet” adaptation but rather the product of an integrated suite of morphological traits, protective metabolites, specialized proteins, and regulatory circuits. These adaptations operate synergistically: the thick extracellular polysaccharide (EPS) sheath and UV-screening pigments shield cells from external hazards [9], while a battery of antioxidants, water stress proteins, high-light inducible proteins (HLIPs), and dynamic carotenoid proteins preserve vital cellular structures [10]. Importantly, *N. flagelliforme* anticipates environmental fluctuations by employing light cues and possibly circadian regulation to proactively prepare for desiccation each day [3]. The organism’s large and complex genome encodes this foresight, with expanded gene families and regulatory elements specifically adapted to life at the margins of habitability.

Research on *N. flagelliforme* has progressed significantly in the last decade. Early studies in the 1990s provided descriptive insights into its drought-hardiness and slow growth, depicting the organism as a “hairy alga” with unusual physiological traits [2]. Contemporary genomic and molecular studies have provided mechanistic depth, uncovering the molecular foundations of *N. flagelliforme*’s stress tolerance. These advancements include the identification of a novel UV-B-inducible sunscreen, an ornithine-containing mycosporine-like amino acid (MAA) [9], and its transcriptional regulator; the elucidation of a red light signaling pathway that links the diurnal cycle to cellular stress readiness [59]; and the characterization of stress-specific proteins such as the dehydration-induced NfDnaK2 chaperone [53] and the cold-responsive CsrR1 protein with dual stress tolerance functions [7]. These findings emphasize that *N. flagelliforme*’s stress tolerance is an actively regulated phenotype, not a passive survival mechanism. The evolution of expanded *hlip* and orange carotenoid protein (OCP) families in *Nostoc* highlights the utilization of gene duplication and divergence to address challenges of extreme light and oxygen stress [5,10,52]. Similarly, the proliferation of regulatory genes (such as sigma factors and two-component systems) in its genome reflects an ongoing evolutionary arms race to monitor and respond to environmental nuances [5,10,53,59].

Despite these advancements, significant knowledge gaps remain. One primary gap is identifying the sensors involved in detecting water availability. How does *N. flagelliforme* initially detect the onset of drying? Deciphering the signal transduction pathways from physical water loss to genetic response, such as understanding what triggers Hrf1 to lift repression or which kinase activates OrrA, remains a priority for future research. Additionally, one traditional methodological challenge has been the difficulty of maintaining and manipulating *N. flagelliforme* in laboratory cultures. Long considered “unculturable” axenically, or very slow-growing in liquid media, recent success in applying CRISPR-based gene editing in *N. flagelliforme* has demonstrated that these hurdles can be overcome [5,9]. Continued development of genetic tools will enable researchers to create targeted knockouts or reporter constructs to directly test gene function and regulatory interactions. For example, generating a double mutant in *orrA* and *hrf1* could reveal potential cross-talk between UV and desiccation responses, while overexpressing an sRNA predicted to bind *psbA* mRNA could confirm its role in recovery. Such experiments will enrich our understanding and validate models built from omics data.

From an applied perspective, insights gleaned from *N. flagelliforme* hold considerable promise for biotechnology and agriculture. The idea of engineering drought-tolerant crops by transferring cyanobacterial stress genes is already under exploration. Genes like the *mys* MAA biosynthesis cluster could potentially enhance stress resistance when expressed in plant chloroplasts or other microbes. The use of *N. flagelliforme* in desert soil restoration is another exciting avenue. For instance, Chinese researchers have trialed inoculating crust-forming cyanobacteria to combat desertification. A deeper understanding of *N. flagelliforme*’s life cycle and requirements will inform how to cultivate it at scale or deploy it effectively as a bio-tool for stabilizing soils. Furthermore, *N. flagelliforme* produces unique metabolites, such as MAAs, polysaccharides, and possibly antimicrobial compounds, that could have pharmaceutical or cosmetic applications, particularly as natural UV protectants.

In conclusion, the story of *N. flagelliforme* is one of resilient life at the edge: showcasing the versatility of cyanobacteria and the power of evolutionary innovation. From its morphological adaptations to its molecular strategies, *N. flagelliforme* exemplifies how organisms can adapt to severe and fluctuating conditions. As research tools improve and global interest in stress biology grows—driven, in part, by climate change making extreme environmental conditions more common—*N. flagelliforme* will likely receive increased attention. Many of its strategies such as the formation of protective extracellular matrices, the use of anticipatory signals, and the deployment of multilevel gene regulation, are relevant to other biological contexts and even to engineering systems. By continuing to unravel the secrets of *N. flagelliforme*, we not only satisfy scientific curiosity about how *N. flagelliforme* survives but also gain valuable blueprints for designing stress-resistant life forms and preserving fragile ecosystems. In the spirit of the organism’s Chinese name, “facai” meaning “to prosper”, the knowledge derived from *N. flagelliforme* research may help us cultivate prosperity in environments that today seem too harsh to sustain productivity or biodiversity.

## Data Availability

All mentioned genes and proteins are included in the Supplementary Materials.

## Supplementary Materials

The following supporting information can be downloaded at: https://www.mdpi.com/article/10.3390/plants14111582/s1, Table S1: All mentioned genes and proteins in the Manuscript.
